# Health related quality of life after combined hormone replacement therapy: randomised controlled trial

**DOI:** 10.1136/bmj.a1190

**Published:** 2008-08-21

**Authors:** Amanda J Welton, Madge R Vickers, Joseph Kim, Deborah Ford, Beverley A Lawton, Alastair H MacLennan, Sarah K Meredith, Jeannett Martin, Tom W Meade

**Affiliations:** 1 MRC General Practice Research Framework, Stephenson House, London NW1 2ND; 2 London School of Hygiene and Tropical Medicine, London; 3 MRC Clinical Trials Unit, London; 4 Women’s Health Research Centre, Department of Primary Health Care and General Practice, Wellington School of Medicine and Health Sciences, New Zealand; 5 Discipline of Obstetrics and Gynaecology, School of Paediatrics and Reproductive Medicine, University of Adelaide, Women’s and Children’s Hospital, Adelaide SA 5005, Australia

## Abstract

**Objective** To assess the effect of combined hormone replacement therapy (HRT) on health related quality of life.

**Design** Randomised placebo controlled double blind trial.

**Setting** General practices in United Kingdom (384), Australia (94), and New Zealand (24).

**Participants** Postmenopausal women aged 50-69 at randomisation; 3721 women with a uterus were randomised to combined oestrogen and progestogen (n=1862) or placebo (n=1859). Data on health related quality of life at one year were available from 1043 and 1087 women, respectively.

**Interventions** Conjugated equine oestrogen 0.625 mg plus medroxyprogesterone acetate 2.5/5.0 mg or matched placebo orally daily for one year.

**Main outcome measures** Health related quality of life and psychological wellbeing as measured by the women’s health questionnaire. Changes in emotional and physical menopausal symptoms as measured by a symptoms questionnaire and depression by the Centre for Epidemiological Studies depression scale (CES-D). Overall health related quality of life and overall quality of life as measured by the European quality of life instrument (EuroQol) and visual analogue scale, respectively.

**Results** After one year small but significant improvements were observed in three of nine components of the women’s health questionnaire for those taking combined HRT compared with those taking placebo: vasomotor symptoms (P<0.001), sexual functioning (P<0.001), and sleep problems (P<0.001). Significantly fewer women in the combined HRT group reported hot flushes (P<0.001), night sweats (P<0.001), aching joints and muscles (P=0.001), insomnia (P<0.001), and vaginal dryness (P<0.001) than in the placebo group, but greater proportions reported breast tenderness (P<0.001) or vaginal discharge (P<0.001). Hot flushes were experienced in the combined HRT and placebo groups by 30% and 29% at trial entry and 9% and 25% at one year, respectively. No significant differences in other menopausal symptoms, depression, or overall quality of life were observed at one year.

**Conclusions** Combined HRT started many years after the menopause can improve health related quality of life.

**Trial registration** ISRCTN 63718836.

## Introduction

The quality of an individual’s life is an important end point in medical and health outcomes research.^1^ In many circumstances quality of survival is as important as quantity.^2^ Health related quality of life is a reflection of the way patients feel or function. There is debate about the effect of hormone replacement therapy (HRT) on health related quality of life, though it has been shown to improve general quality of life through the reduction of both the number and severity of menopausal symptoms.^3^ ^4^

The perception of the risks and benefits of HRT and its timing has changed dramatically since the publication of the women’s health initiative trial in 2002.^5^ That study of women initiating HRT on average 13 years after menopause did not find long term benefits on cardiovascular outcomes, previously suggested by observational studies of women initiating HRT near menopause. The impact of HRT on patient centred outcomes such as health related quality of life was questioned.^6^ ^7^ Although the study reported a significant benefit of combined oestrogen and progestogen (combined HRT) in health related quality of life related to sleep disturbance, physical functioning, and bodily pain, the differences were small, and for conjugated equine oestrogen no effect was found in women who had had a hysterectomy.^7^ The smaller heart and oestrogen/progestin replacement study (HERS) found that menopausal symptoms modified the effect of HRT on health related quality of life: women without flushing had greater declines in health related quality of life related to physical measures, while those with flushing experienced improvements in health related quality of life related to emotional measures.^8^ Previous studies have suggested that HRT has an important positive impact on various symptoms of the menopause and thus overall health related quality of life.^4^ ^9^

The two trials used generic instruments for measuring quality of life, which are less sensitive to changes in health related quality of life resulting from a particular outcome or condition. Improvements in health related quality of life attributable to the presence or absence of postmenopausal symptoms might thus have been overlooked as generic instruments do not contain specific items on postmenopausal symptoms.^10^ Condition specific instruments might be more appropriate for use in a randomised controlled trial.^11^

The women’s international study of long duration oestrogen after the menopause (WISDOM) aimed to evaluate the long term benefits and risks of HRT. WISDOM was a placebo controlled, double blind randomised trial of HRT in postmenopausal women. It aimed to randomise 22 300 postmenopausal women aged 50-69 from primary care in the United Kingdom, Australia, and New Zealand to HRT or placebo for a median of 10 years. In addition to the main clinical outcomes of cardiovascular disease, fractures, and breast cancer, WISDOM included a detailed assessment of the impact of HRT on health related quality of life. Recruitment began in 1999 and continued until October 2002, when the trial was closed after the announcement that the women’s health initiative had been terminated early because the risks of combined HRT seemed to outweigh the benefits in that population.^5^

We present findings from WISDOM for women with an intact uterus or subtotal hysterectomy who were randomised to combined HRT or placebo, focusing on health related quality of life measured at one year follow-up using both condition specific measures designed specifically for postmenopausal women and generic measures.

## Methods

Full details of the protocol, methods, and study participants are described elsewhere.^12^ ^13^ ^14^ Participants were recruited from general practices in the UK, Australia, and New Zealand. Postmenopausal women aged 50-69 at randomisation with an intact uterus or subtotal hysterectomy were randomised to combined HRT or placebo.^14^ Combined HRT was oral conjugated equine oestrogen 0.625 mg daily, plus oral medroxyprogesterone acetate 2.5/5.0 mg daily (Prempro, Wyeth Ayerst, USA).

The trial was designed to treat participants for a median of 10 years with scheduled visits at four weeks, 14 weeks, 27 weeks, 40 weeks, and 52 weeks, with annual visits thereafter. All participants were encouraged to continue attending annual interviews whether or not they had stopped taking study treatment. When the study closed early the median follow-up time was one year. After closure participants were asked to continue taking study medication until they were able to attend a clinic for a final visit, which included the interview conducted annually, irrespective of a participant’s time in the trial. This report is limited to data collected a year after randomisation as information on health related quality of life was available on less than half of all study participants by the end of the second year.

### Outcome measures

The women’s health questionnaire is designed to assess physical and emotional wellbeing in middle aged women.^15^ ^16^ We used a modified version comprising a five item self esteem scale and items intended to capture eight components of women’s health: depressed mood (seven items), somatic symptoms (seven items), memory and concentration (three items), vasomotor symptoms (two items), anxiety or fears (four items), sexual functioning (three items), sleep problems (three items), and menstrual symptoms (four items).^17^ Each item was rated on a four point scale: “yes, definitely”(1), “yes, sometimes” (2), “no, not much” (3), and “no, not at all” (4), and this was reduced to a binary scale—that is, 1 and 2 (coded 0) *v* 3 and 4 (coded 1) for scoring. For each domain we computed the mean score for the corresponding items, thus the higher the score, the better the quality of life on that specific domain.

We assessed the prevalence of individual symptoms related to menopause using a 28 item symptom questionnaire that asked about the presence of symptoms occurring in the past four weeks. Depression was assessed with the Center for Epidemiologic Studies depression scale (CES-D), a 20 item self report depression scale designed to measure the presence and severity of depressive symptomatology in the general population.^18^ ^19^ Patients are presented with 20 statements and asked to indicate how they have been feeling using four response options from “rarely or none of the time” to “most or all of the time.” Responses are scored from 0 to 3, where higher scores indicate higher frequency (score ranges from 0 to 60). A higher overall score indicates more severe depression; scores above 16 are generally considered high.

The European quality of life instrument (EuroQoL) is a two part generic measure of health related quality of life that has been validated for use in clinical trials.^20^ It consists of the EQ visual analogue scale (EQ-VAS) and an overall health classification index (EQ-5D).^21^ The 10 centimetre EQ visual analogue scale ranged from 0 (“worst imaginable health state”) to 100 (“best imaginable health state”); participants are asked to grade their current state of health (as opposed to the visual analogue scale in which patients rate their overall quality of life, not their health). The six item health classification index assessed five unique dimensions of health related quality of life: mobility, self care, usual activities, pain/discomfort, and anxiety/depression. Each dimension is measured on a three point ordinal scale (that is, “no problem,” “some problem,” and “extreme problem”) such that a higher score corresponds to a worse state of health. Participants are then effectively located into one of 243 possible health states for which a global utility score is assigned. The utility score has been previously determined based on the preferences of a sample of 3395 individuals, representative of the UK general population.^22^ A utility score of 1 corresponds to the highest possible degree of health related quality of life, and score of 0 is compatible with a level equivalent to death. A negative utility score corresponds to a state that is worse than death. The EuroQoL has been previously validated in numerous different settings.^23^ ^24^ ^25^

The visual analogue scale is a single item generic measure designed to assess all aspects of life, not only those that are health related.^26^ ^27^ On a 10 centimetre scale from 0 (“might as well be dead”) to 100 (“perfect quality of life”), participants were asked “Which number best represents your overall quality of life over the past two weeks?”^28^

All measures were administered at baseline and at annual visits. The visual analogue scale and symptoms questionnaire were also administered at intermediate follow-up visits (that is, four weeks, 14 weeks, 27 weeks, and 40 weeks).

### Statistical analysis

All analyses were intention to treat. Treatments were compared with multiple or logistic regression analyses, including the measure of interest at baseline as a covariate to adjust for any differences between treatment groups at baseline. The geometric mean value was used in the analysis of the CES-D data because the distribution was right skewed. We evaluated the possible modifying effect of baseline vasomotor symptoms on effects of treatment at one year in health related quality of life by fitting an interaction term(s). Significance of the effect of combined HRT on individual outcomes at one year was judged by Bonferroni corrected α levels of 0.001 (about 0.05/41). Interaction tests were performed only if we identified a significant main effect and were used only to look at associations with baseline vasomotor symptoms. Analyses of visual analogue scales were performed at each time point and within subgroups with no adjustment for multiple testing. Text and tables show unadjusted P values. All statistical analyses were performed with Stata (version 9.2, College Station, TX).

## Results

At the end of the trial 3721 women with an intact uterus or subtotal hysterectomy had been randomised, 1862 to combined HRT and 1859 to placebo.^13^ The figure shows the flow of women after randomisation for this report.[Fig fig1] We excluded women who had been in the trial less than 40 weeks at trial closure in October 2002 or who had died within one year of randomisation (total 671 combined HRT; 666 placebo). Of the remainder, 148 and 106 women randomised to combined HRT and placebo, respectively, did not attend their one year interview and cannot contribute to the primary analyses (figure).[Fig fig1] Mean age at randomisation in the 2130 women in the primary analyses was 63.8 years (SD 4.4). The median time between randomisation and one year interview was 368 days (90% interviews were 331-456 days after randomisation), and 21% of one year interviews (21% combined HRT; 20% placebo) were completed after trial closure.

**Figure fig1:**
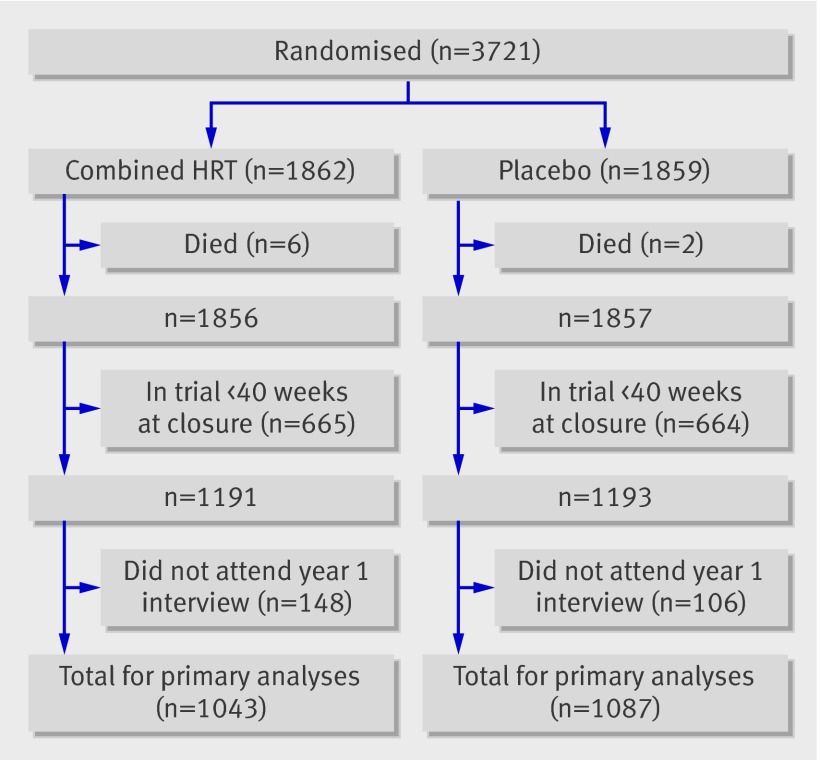
Flow of participants after randomisation

At the first annual medical 290 (28%) women randomised to combined HRT were no longer taking trial treatment (214 had stopped permanently and 76 were on a temporary interruption of treatment). Of the 214 who stopped combined HRT permanently, 91 did so in the first 14 weeks and 177 did so in the first 27 weeks. Overall, trial treatment was supplied for 79% of follow-up time between randomisation and first annual medical. Correspondingly, at the first annual medical, 141 (13%) in the placebo group were not taking trial treatment (89 had stopped permanently; 52 were on a temporary interruption); trial treatment was supplied for 92% of follow-up time. The most common reasons for permanent discontinuation of combined HRT were vaginal bleeding (68/214, 32%) and breast tenderness (28/214, 13%). Of the patients who were not taking trial treatment at the first annual medical, 174 (60%) in the combined HRT group and 10 (7%) in the placebo group had previously reported bleeding to some degree, including spotting. Of those taking trial treatment at one year, 265 (37%) in the combined HRT group had reported bleeding at some time during the first year, including 65 (9%) since their last visit. Corresponding proportions in the placebo group were 36 (4%), including 9 (1%) since their last visit.

We reviewed the data on the women who did not attend their one year interview and therefore could not be included in the primary analyses. Most were known to have discontinued trial treatment: 132/148 (89%) women randomised to combined HRT stopped treatment permanently before one year (n=118) or were on a temporary interruption of treatment at one year (n=14) and did not restart treatment before study closure; in the placebo group 84/106 (79%) had discontinued trial treatment (68 permanently and 16 on a temporary interruption). As in the women who attended the one year interview, the most common reasons for permanent discontinuation of combined HRT were vaginal bleeding (42/118, 36%) and breast tenderness (13/118, 11%.).

### Women’s health questionnaire 

According to assessment with the women’s health questionnaire, participants in the combined HRT group experienced significant improvements in vasomotor symptoms compared with the placebo group at one year (difference between treatment groups, adjusted for baseline score, 0.09, 95% confidence interval 0.07 to 0.12), P<0.001) (table 1). Treatment differences were more marked in those with more severe baseline symptoms (P<0.001). Participants also reported small but significant improvements related to sexual functioning (difference between treatment groups, adjusted for baseline score, 0.05, 0.02 to 0.08, P<0.001) and sleep problems (difference between treatment groups, adjusted for baseline score, 0.05, 0.02 to 0.07, P<0.001). Although sleep problems at one year were marginally higher in those with baseline vasomotor symptoms, there was no evidence for a difference in treatment benefit by baseline vasomotor symptoms (P=0.78). Sexual function at one year was not associated with baseline vasomotor symptoms (P=0.78).

**Table 1 tbl1:** Mean (SE) scores on health related quality of life as measured with women’s health questionnaire by treatment group

Component	Baseline			One year		Adjusted† difference at one year (95% CI)	P value
	Combined HRT (n=1043*)	Placebo (n=1087*)		Combined HRT (n=1043*)	Placebo (n=1087)*		
Depression	0.803 (0.004)	0.797 (0.004)		0.803 (0.004)	0.805 (0.004)	0.00 (−0.01 to 0.01)	0.39
Somatic	0.755 (0.006)	0.764 (0.006)		0.775 (0.006)	0.781 (0.006)	0.00 (−0.02 to 0.01)	0.80
Memory	0.730 (0.010)	0.721 (0.009)		0.759 (0.009)	0.757 (0.009)	0.00 (−0.02 to 0.02)	0.88
Vasomotor	0.766 (0.012)	0.771 (0.011)		0.926 (0.007)	0.833 (0.010)	0.09 (0.07 to 0.12)	<0.001‡
Anxiety	0.882 (0.006)	0.882 (0.006)		0.894 (0.006)	0.904 (0.006)	0.00 (−0.02 to 0.00)	0.18
Sexual	0.679 (0.012)	0.679 (0.013)		0.764 (0.0110	0.721 (0.012)	0.05 (0.02 to 0.08)	<0.001‡
Sleep	0.637 (0.010)	0.657 (0.010)		0.740 (0.009)	0.703 (0.009)	0.05 (0.02 to 0.07)	<0.001‡
Menstrual	0.906 (0.005)	0.905 (0.005)		0.905 (0.005)	0.907 (0.005)	0.00 (−0.01 to 0.01)	0.77
Esteem	0.546 (0.004)	0.544 (0.004)		0.559 (0.004)	0.553 (0.004)	0.00 (−0.01 to 0.02)	0.40

*No in each group at each time point for any measure except sexual function (n=591 for cHRT group (588 at one year) and 580 for placebo group (569 at one year) for women who attended interview).

†Adjusted for baseline score.

‡Significant at Bonferroni corrected α level of 0.001; actual unadjusted P values presented.

### Menopausal symptoms

Table 2 shows the distribution of individual menopausal symptoms by treatment group. At one year, with adjustment for differences at baseline, participants randomised to combined HRT experienced fewer vasomotor symptoms, including hot flushes (9% *v* 25%, P<0.001) and night sweats (14% *v* 23%, P<0.001) than those randomised to placebo. They were also less likely to report symptoms of aching joints and muscles (57% *v* 63%, P=0.001), insomnia (35% *v* 41%, P<0.001), and vaginal dryness (14% *v* 19%, P<0.001). Bloating was marginally less prevalent in the combined HRT group than the placebo group (21% *v* 24%, P=0.005), but significance was not achieved when we allowed for multiple testing. The combined HRT group reported higher rates of breast tenderness (16% *v* 7%, P<0.001) and vaginal discharge (14% *v* 5%, P<0.001) than the placebo group.

**Table 2 tbl2:** Prevalence of symptoms related to menopause. Figures are numbers (percentages) of women

Symptom	Baseline			One year		Adjusted† difference at one year (95% CI)	P value§
	Combined HRT (n=1041*)	Placebo (n=1085*)		Combined HRT (n=1043*)	Placebo (n=1087*)		
Hot flushes	317 (30)	311 (29)		98 (9)	269 (25)	−15 (−18 to −12)	<0.001‡
Night sweats	283 (27)	281 (26)		145 (14)	252 (23)	−9 (−13 to −6)	<0.001‡
Insomnia	471 (45)	472 (44)		367 (35)	450 (41)	−6 (−10 to −2)	<0.001‡
Feeling depressed	195 (19)	207 (19)		234 (22)	256 (24)	−1 (−5 to 2)	0.6
Feeling anxious	293 (28)	284 (26)		300 (29)	313 (29)	0 (−4 to 4)	0.7
Dizziness	117 (11)	137 (13)		133 (13)	143 (13)	0 (−3 to 2)	1
Aching joints or muscles	659 (63)	680 (63)		592 (57)	688 (63)	−7 (−11 to −2)	<0.001‡
Tiredness	523 (50)	516 (48)		556 (53)	554 (51)	2 (−2 to 7)	0.5
Headache	264 (25)	288 (27)		268 (26)	273 (25)	1 (−3 to 4)	0.6
Migraine	44 (4)	56 (5)		51 (5)	60 (6)	−1 (−3 to 1)	0.8
Irritability/mood swings	181 (17)	183 (17)		186 (18)	220 (20)	−2 (−6 to 1)	0.1
Heart racing or skipping beats	114 (11)	112 (10)		149 (14)	128 (12)	2 (0 to 5)	0.1
Dry skin or scaling	319 (31)	332 (31)		304 (29)	326 (30)	−1 (−5 to 3)	0.6
Vaginal or genital dryness	249 (24)	244 (22)		150 (14)	211 (19)	−5 (−8 to −2)	<0.001‡
Vaginal or genital itching	115 (11)	101 (9)		121 (12)	118 (11)	1 (−2 to 3)	0.9
Vaginal or genital discharge	58 (6)	48 (4)		151 (14)	55 (5)	9 (7 to 12)	<0.001‡
Pain or burning while urinating	31 (3)	31 (3)		36 (3)	39 (4)	0 (−2 to 1)	0.8
Breast tenderness	65 (6)	69 (6)		164 (16)	76 (7)	9 (6 to 11)	<0.001‡
Leg cramps in one leg	96 (9)	108 (10)		122 (12)	113 (10)	1 (−1 to 4)	0.3
Leg cramps in both legs	169 (16)	167 (15)		227 (22)	205 (19)	3 (−1 to 6)	0.1
Swelling in one leg	45 (4)	35 (3)		44 (4)	31 (3)	1 (0 to 3)	0.2
Swelling in both legs	96 (9)	80 (7)		82 (8)	98 (9)	−1 (−4 to 1)	0.07
Nausea	85 (8)	87 (8)		84 (8)	91 (8)	0 (−3 to 2)	0.8
Abdominal cramps	70 (7)	76 (7)		81 (8)	95 (9)	−1 (−3 to 1)	0.4
Bloating	235 (23)	217 (20)		215 (21)	260 (24)	−3 (−7 to 0)	0.005
Skin rash/itching	185 (18)	166 (15)		187 (18)	177 (16)	2 (−2 to 5)	0.6
Crawling feelings under skin	83 (8)	81 (7)		77 (7)	76 (7)	0 (−2 to 3)	0.8
Trouble seeing not corrected by glasses/contact lenses	46 (4)	54 (5)		51 (5)	64 (6)	−1 (−3 to 1)	0.4

*No of women with available data.

†Adjusted for baseline presence of symptom.

‡Significant at Bonferroni corrected α level of 0.001; actual unadjusted P values presented.

In a secondary analysis of symptoms at one year we carried forward the last observation after entry for all women without a one year interview who had been in the trial at least 40 weeks at closure (allowing us to include 99% of women in both treatment groups). Overall results (not shown) were similar, although the proportion of women reporting breast tenderness in the combined HRT group at their last visit who had not attended a one year interview was somewhat higher (48%, 64/133) than in the group interviewed at one year (16%, 164/1043).

### Vasomotor symptom interactions

We explored interactions between the treatment effect at one year and baseline reporting of hot flushes or night sweats. The benefit of combined HRT was greater in those who had symptoms at baseline: at one year in the combined HRT group 22% of those who had reported hot flushes at baseline were still having hot flushes compared with 65% in the placebo group, while in women who had not reported hot flushes at baseline corresponding proportions were 4% and 9%, respectively (interaction P=0.002). Similarly, only 33% of women randomised to combined HRT who reported night sweats at baseline were still having them at one year compared with 62% of women randomised to placebo; corresponding proportions of those who had not reported night sweats at baseline were 7% and 10% (interaction P=0.002). We investigated whether the impact of combined HRT on other symptoms varied according to baseline vasomotor symptoms but found no interaction for aching joints and muscles, insomnia, vaginal dryness, breast tenderness, or vaginal discharge.

### Depression

Assessment of depression with the CES-D at one year showed no significant difference between the combined HRT (median 3, interquartile range 0-7) and placebo groups (median 3, 1-8; P=0.51 for difference, adjusted for baseline score) and no difference in the proportion of individuals who experienced high depressive scores (CES-D >16 units) between the two treatment groups (8% *v* 9%; P=0.51, adjusted for baseline).

### Quality of life

There were no differences in self assessed health measured by the EQ visual analogue scale at one year follow-up (table 3). Participants randomised to combined HRT had a marginally higher health classification index score compared with those randomised to placebo (difference between treatment groups adjusted for baseline score 0.016 units, 95% confidence interval 0.003 to 0.028, P=0.02) but the difference was not significant when we applied the Bonferroni correction.

**Table 3 tbl3:** EuroQoL scores by treatment group. Figures are means (SE)

	Baseline			One year			Adjusted† difference at one year (95% CI)	P value
	Combined HRT (n=1042*)	Placebo (n=1083*)		Combined HRT (n=1043*)		Placebo (n=1087*)		
EQ-VAS	79.2 (0.4)	79.4 (0.4)		77.9 (0.5)		78.5 (0.4)	−0.59 (−1.66 to 0.47)	0.28
EQ-5D	0.877 (0.005)	0.874 (0.005)		0.888 (0.005)		0.870 (0.005)	0.016 (0.003 to 0.028)	0.02

*No of women with available data.

†Adjusted for baseline score.

### Generic visual analogue scale

At four weeks women in the combined HRT group had a slightly lower overall quality of life than those in the placebo group (difference, adjusted for baseline values, −1.6 units, −2.7 to −0.4, P=0.006, table 4). This difference was reduced at 14 weeks (P=0.03), and there were no differences between the combined HRT and the placebo groups after 14 weeks. We lacked power to investigate the extent to which the early reduction in visual analogue score associated with combined HRT declined because women who experienced a negative impact of combined HRT on quality of life stopped trial treatment early. The difference between treatment groups at four weeks was somewhat greater in women who stopped trial treatment before their first annual medical (difference −2.6 units, −5.4 to 0.2) than in women who were on trial treatment at one year (difference −1.0 units, −2.2 to 0.2, P=0.3 for difference).

**Table 4 tbl4:** Differences in generic visual analogue scale for overall quality of life between treatment groups

	Combined HRT			Placebo		Mean (95% CI) difference between treatment groups*	P value*
	No with data	Mean (SE)		No with data	Mean (SE)		
Baseline	1042	78.1 (0.4)		1083	78.1 (0.4)	—	—
4 weeks	1009	74.9 (0.5)		1049	76.3 (0.4)	−1.6 (−2.7 to −0.4)	0.006
14 weeks	951	74.9 (0.5)		1051	76.0 (0.4)	−1.3 (−2.4 to −0.1)	0.03
27 weeks	878	75.7 (0.5)		1035	75.6 (0.5)	−0.2 (−1.4 to 1.0)	0.78
40 weeks	827	75.6 (0.5)		983	75.7 (0.5)	−0.2 (−1.4 to 1.1)	0.79
52 weeks	1041	77.1 (0.5)		1086	77.4 (0.4)	−0.5 (−1.6 to 0.7)	0.42

*Adjusted for baseline score.

We repeated analyses using all available data at each interview (on participants randomised at least 40 weeks before closure) and found similar results (treatment difference at four weeks −1.35 units, P=0.012; 1137 patients in the combined HRT group and 1141 patients in the placebo group).

## Discussion

In this randomised controlled trial women who started taking combined HRT many years after menopause experienced improved sleep and reduced vasomotor symptoms. They also reported fewer aching joints and muscles, less vaginal dryness, and improved sexual functioning, but breast tenderness and vaginal discharge increased. The beneficial changes in sleep and sexual functioning were independent of the presence of baseline vasomotor symptoms (flushes or night sweats). For most other condition specific measures there were no differences between placebo and combined HRT groups.

These improvements in health related quality of life for postmenopausal women were detected by using the outcome specific women’s health questionnaire and a symptoms questionnaire. There were no significant changes, however, in health related quality of life identified with the generic EuroQoL or in quality of life with the visual analogue scale. The former condition specific questionnaires might be more sensitive to menopausal changes influencing quality of life.

### Comparison with published data

The improvements seen in WISDOM are consistent with results from the women’s health initiative, showing improvement in vasomotor symptoms, sleep, and bodily pain after one year of combined HRT.^6^ Self reported sleep problems have been associated with both the perimenopause and postmenopause. Disturbed sleep can result in daytime sleepiness, which in turn can increase the risk of accidents, lead to underperformance in the workplace, and affect the family and quality of life.^29^ Inadequate sleep is also associated with an increased risk of illnesses such as obesity, diabetes, hypertension, and cardiovascular disease.^30^ ^31^ ^32^ Reducing sleep deprivation might therefore have considerable health benefits.

Aches and stiff joints are commonly reported by menopausal women, and self reported arthritis is associated with menopausal status.^33^ A follow-up study with participants in the women’s health initiative that looked at joint symptoms showed a higher prevalence of pain or stiffness in those women who stopped taking combined HRT compared with placebo.^34^ Animal models also suggest that oestrogen has an anaesthetic role and might prevent cartilage erosion such as occurs in osteoarthritis.^35^ ^36^ ^37^

For women taking combined HRT there was a significant improvement on the three item sexual functioning domain of the menopause specific women’s health questionnaire. It is difficult to compare this finding with the women’s health initiative, particularly as it has been argued that the single question screen for sexual satisfaction used in the women’s health initiative was inadequate.^38^ The WISDOM result is consistent with the recent findings from the COGENT study, a randomised controlled trial using combined HRT (conjugated equine oestrogen 0.625 mg/medroxyprogesterone acetate 2.5 mg ) for four months, which showed women treated with combined HRT had an increase in sexual thoughts and interest compared with placebo.^39^ These women were aged 45-55 years. The improvement in sexual functioning noted in the older age group studied in WISDOM warrants further investigation.

Neither WISDOM nor the women’s health initiative found an effect of combined HRT on depression measured by CES-D. In WISDOM, the depression component of the women’s health questionnaire also showed no difference between combined HRT and placebo groups. Two randomised controlled trials involving older women found no effect of HRT on depression.^40^ ^41^ Two small randomised controlled trials have shown a benefit of oestradiol in depressed perimenopausal women.^42^ ^43^ It is possible that any antidepressant effect of oestrogen seen in women at or near the menopause might be related to reduction of menopausal symptoms.

About 22% of all women in the trial complained of bloating at trial entry. This symptom might reflect other common postmenopausal conditions such as irritable bowel syndrome, peritoneal adhesions, and increasing central obesity. While HRT has sometimes been blamed for bloating in individual patients or in uncontrolled series, in this study combined HRT did not worsen bloating.

### Weaknesses of study

Loss to follow-up and considerable discontinuation of study medication, particularly in the combined HRT group, were weaknesses that might have introduced selection bias. Because of premature closure of the trial around 36% of women in both treatment groups had not reached 40 weeks of treatment at closure and were excluded from all analyses in this paper. One year data were not available for 12% of women randomised to combined HRT and 9% of women randomised to placebo at least 40 weeks before trial closure. Most participants who did not attend a one year interview had stopped study treatment, so it is likely that their inclusion in an intention to treat analysis would have reduced the treatment effects we observed. Non-attenders at one year, however, might have had a different symptom profile and overall quality of life than attenders, and therefore some degree selection bias is possible.

Participants who attended a one year interview but were no longer taking treatment in the combined HRT group experienced more side effects early in the trial than those who remained on treatment, specifically bleeding and breast tenderness. Many similar combined HRT regimens are associated with early bleeding that mostly disappears over the first months of treatment.^44^ Overall, the effect of early bleeding and breast tenderness with combined HRT, causing women to preferentially discontinue treatment, might largely explain the reduction in quality of life as assessed by the visual analogue scale score at 4 and 14 weeks of treatment. Even in those women who remained taking treatment, however, there was a small, non-significant drop in quality of life at four weeks in the combined HRT group compared with placebo, suggesting a small true early adverse effect. Our study lacked power to show whether the effect differs in those who keep taking treatment and those who stop.

In the placebo group some of those who stopped the study medication might have started HRT outside the trial, although of the 89 patients on placebo who stopped taking trial treatment permanently, only five did so because they wanted to take HRT. It is likely that both side effects and benefits of combined HRT are greater than estimated in patients who keep taking treatment.

The impact of the early closure of WISDOM is difficult to predict. Over a fifth (21%) of interviews were after closure, and participants’ responses might have been influenced by the publicity surrounding closure and by the knowledge that the trial was closing.

While the size of the differences in health related quality of life was small, the presence of improvements in sleep, aching joints, and relief of menopause symptoms is consistent across other studies, including the women’s health initiative, and these might be important benefits for many symptomatic women. The WISDOM results are limited by the relatively short duration of follow-up and therefore we cannot say whether these benefits would be maintained over a longer treatment time or whether symptoms would return after cessation of treatment as was seen for joint pains in the women’s health initiative.^34^

### Relevance of findings

The results of WISDOM are clinically relevant for doctors treating postmenopausal women with relevant symptoms including vasomotor symptoms, sleep disturbance, and aching joints and muscles. When women start combined HRT many years after the menopause they must balance the risk of increased cardiac events, venous thromboembolism, and breast cancer against possible benefits on health related quality of life.^5^ ^14^ They must also consider possible early side effects of combined HRT such as breast tenderness, vaginal discharge, and uterine bleeding, which usually resolve with time or dose titration.^14^ These side effects are common hyperoestrogenic symptoms and might reflect the fixed dose regimen in this trial, which was relatively high for women aged over 60.

These results should be interpreted with caution, as the presence of statistical significance does not imply clinical significance.^45^ Differences between treatments for most of the health related quality of life measures in this study were small, with the exception of improvements related to vasomotor symptoms, and most measures were not affected by combined HRT. Conversely, because WISDOM and previous randomised controlled trials such as the women’s health initiative and the heart and oestrogen/progestin replacement study (HERS) included women well past the menopause who were mainly asymptomatic, the results of these trials are likely to underestimate the positive effects of combined HRT on health related quality of life for women with symptoms. There is much background literature and guidelines that support the premise that women with debilitating menopause symptoms will have the most benefit in health related quality of life from treatment with combined HRT.^46^ ^47^ This is an important message to convey to women with severe menopausal symptoms. Additionally, for younger women, recent data suggest a more favourable risk profile for combined HRT when it is started within a few years of menopause compared with many years after.^48^ ^49^ ^50^

The women’s health initiative (and to a lesser extent WISDOM) have helped to answer the question as to whether some chronic diseases could be prevented or ameliorated when combined HRT is started many years after the menopause. Chronic disease outcomes should not be considered in isolation from outcomes of health related quality of life in this age group. 

### Relevance for public health

The public health implication of our findings is that combined HRT might improve some aspects of health related quality of life in the late menopausal phase in women with and without classic vasomotor menopausal symptoms. Combined HRT improves sleep, aches and pains, and sexual functioning. These gains can now be factored in to a woman’s choice to use combined HRT. This benefit must be weighed against the overall short and long term risks, which must be individualised for women based on years since menopause, medical history, and chosen regimen. Further research with appropriate measures is needed to assess more fully the impact of combined HRT on all aspects of health related quality of life for postmenopausal women. In light of the WISDOM findings on quality of life consideration should be given to revisiting HRT guidelines.

**What is already known on this topic**

Combined HRT has a small but significant benefit in health related quality of life related to sleep disturbance, physical functioning, and bodily pain
Menopausal symptoms increase the effect of combined HRT on health related quality of life: women with flushing experienced improvements in health related quality of life related to emotional measures

**What this study adds**

Combined HRT started many years after menopause is associated with improvements in vasomotor symptoms, sexual function, sleep disturbance, aching joints and muscles, insomnia, and vaginal dryness but more women experience breast tenderness and vaginal discharge
Aching joints and muscles, insomnia, and vaginal dryness improve independently of whether participants experience vasomotor symptoms at baseline
Combined HRT improves condition specific but not overall generic measures of health related quality of life at one year

## Notes

We are grateful to all the international general practices and practitioners and women who participated in WISDOM.

Other members of the WISDOM team: M Abdalla, B L DeStavola, statistical analysis, department of epidemiology and population health, London School of Hygiene and Tropical Medicine; P Allen, H Balen, R Bastick, H Brown, K Foulger, S Fox, V Glynn, A Hall, L Hand, A Hill, C Leathem, W Mackinnon, E Marshall, A Williams, regional training nurses; N Collins N, B O’Conner, trial managers; J H Darbyshire, director; M Ghali, P Furness, M Z Islam, software design for electronic data collection and management; K Harrild, statistical analysis and data management; C Knott, L Taylor, implementation of trial protocol and management, research nurse support; M A Walgrove, contributed to protocol, developed manual, nurse support; H C Wilkes, statistical design, C-Q Zhu, statistician; S R Zuhrie, development of trial protocol and medical advice; MRC General Practice Research Framework, London; E Griffith, data manager and pharmacy control; P Ryan, co-investigator in South Australia, co-grantee of Australian funding, discipline of public health, University of Adelaide, Australia; P Komesaroff, senior investigator in Victoria, University of Monash, Victoria, Australia; Marley J, senior investigator in NSW and co-grantee of Australian funding, University of Newcastle, New South Wales, Australia; B J Paine, trial coordinator, discipline of obstetrics and gynaecology, University of Adelaide; N P Stocks, medical director, discipline of general practice, University of Adelaide; A Dowell, senior investigator, S Rose, co-investigator, department of primary healthcare and general practice, Wellington School of Medicine and Health Sciences, New Zealand.

Contributors: AJW contributed to the selection of the health related quality of life instruments, was responsible for overseeing the collection of the quality of life data, and contributed to the analysis and interpretation of data and drafting of manuscript. MRV was the WISDOM principal investigator and co-wrote the application for funding with AJW. TWM was responsible for trial design, protocol development, trial procedures, and overall management, contributed to analysis and interpretation of data and drafting of manuscript, and approved the final paper. JK performed statistical analyses and contributed to interpretation of data. DF performed statistical analyses and contributed to interpretation of data and drafting of manuscript. BAL was the principal investigator in New Zealand, contributed to trial design, trial procedures, analyses, and interpretation of data and drafting of manuscript. AHM was the principal investigator in Australia and contributed to trial design, trial procedures, analyses and interpretation of data, and drafting of the manuscript. SKM contributed to revision of the protocol, trial conduct, analysis and interpretation of data, and drafting of the manuscript. JM was responsible for training and coordination of UK research nurses and quality control. TWM was principal investigator in UK and had overall responsibility for studies in the MRC general practice research framework (until retirement in 2001). MRC is guarantor.

Ethical approval: South Thames regional health authority multicentre research ethics committees and relevant local research ethics committees; human research ethics committees for the Universities of Adelaide, Newcastle, and Monash, and the Royal Australian College of General Practitioners’ national research and evaluation ethics committee; Wellington regional ethics committee and Auckland and Canterbury ethics committees.

Provenance and peer review: Not commissioned; externally peer reviewed.

## Notes

**Cite this as:** *BMJ* 2008;337:a1190
